# Fungi in Mangrove: Ecological Importance, Climate Change Impacts, and the Role in Environmental Remediation

**DOI:** 10.3390/microorganisms13040878

**Published:** 2025-04-11

**Authors:** Juliana Britto Martins de Oliveira, Dario Corrêa Junior, Cláudio Ernesto Taveira Parente, Susana Frases

**Affiliations:** 1 Laboratório de Biofísica de Fungos, Instituto de Biofísica Carlos Chagas Filho, Universidade Federal do Rio de Janeiro, Rio de Janeiro 21941-902, Brazil; julianamartins@biof.ufrj.br (J.B.M.d.O.); dariojunior@biof.ufrj.br (D.C.J.); 2 Laboratório de Estudos Ambientais Olaf Malm, Instituto de Biofísica Carlos Chagas Filho, Universidade Federal do Rio de Janeiro, Rio de Janeiro 21941-902, Brazil; cparente@biof.ufrj.br; 3 Rede Micologia RJ, FAPERJ, Rio de Janeiro 21941-902, Brazil

**Keywords:** ecosystems, fungal ecology, sediment microbiota, climate resilience, environmental management, nutrient cycling, environmental stressors, fungal bioremediation

## Abstract

Mangroves are coastal ecosystems of great ecological importance, located in transition areas between marine and terrestrial environments, predominantly found in tropical and subtropical regions. In Brazil, these biomes are present along the entire coastline, playing essential environmental roles such as sediment stabilization, coastal erosion control, and the filtration of nutrients and pollutants. The unique structure of the roots of some mangrove tree species facilitates sediment deposition and organic matter retention, creating favorable conditions for the development of rich and specialized biodiversity, including fungi, bacteria, and other life forms. Furthermore, mangroves serve as important nurseries for many species of fish, crustaceans, and birds, being fundamental to maintaining trophic networks and the local economy, which relies on fishing resources. However, these ecosystems have been significantly impacted by anthropogenic pressures and global climate change. In recent years, the increase in average global temperatures, rising sea levels, changes in precipitation patterns, and ocean acidification have contributed to the degradation of mangroves. Additionally, human activities such as domestic sewage discharge, pollution from organic and inorganic compounds, and alterations in hydrological regimes have accelerated this degradation process. These factors directly affect the biodiversity present in mangrove sediments, including the fungal community, which plays a crucial role in the decomposition of organic matter and nutrient cycling. Fungi, which include various taxonomic groups such as Ascomycota, Basidiomycota, and Zygomycota, are sensitive to changes in environmental conditions, making the study of their diversity and distribution relevant for understanding the impacts of climate change and pollution. In particular, fungal bioremediation has gained significant attention as an effective strategy for mitigating pollution in these sensitive ecosystems. Fungi possess unique abilities to degrade or detoxify environmental pollutants, including heavy metals and organic contaminants, through processes such as biosorption, bioaccumulation, and enzymatic degradation. This bioremediation potential can help restore the ecological balance of mangrove ecosystems and protect their biodiversity from the adverse effects of pollution. Recent studies suggest that changes in temperature, salinity, and the chemical composition of sediments can drastically modify microbial and fungal communities in these environments, influencing the resilience of the ecosystem. The objective of this narrative synthesis is to point out the diversity of fungi present in mangrove sediments, emphasizing how the impacts of climate change and anthropogenic pollution influence the composition and functionality of these communities. By exploring these interactions, including the role of fungal bioremediation in ecosystem restoration, it is expected that this study would provide a solid scientific basis for the conservation of mangroves and the development of strategies to mitigate the environmental impacts on these valuable ecosystems.

## 1. Introduction

Mangroves are coastal ecosystems characterized by vegetation consisting of trees adapted to saline environments in estuaries and coastal regions located in tropical and subtropical regions, providing essential environmental services [1,2]. Their plant diversity includes species such as *Rhizophora mangle*, *Avicennia schaueriana*, *Laguncularia racemosa*, and *Conocarpus erectus*, and they feature wet, saline, and muddy soils rich in nutrients and organic matter, serving as a nursery for a rich diversity of microorganisms, including fungi and bacteria [1,3].

Fungal diversity in the studied mangrove ecosystems varied depending on the geographical location. Previous studies underline the influence of local environmental conditions on fungal communities within mangrove ecosystems [4,5]. In this sense, conducting localized studies is crucial for fully understanding fungal diversity and its ecological roles, as variations in environmental factors such as salinity, organic matter, and hydrodynamics can significantly impact these communities.

Mangroves play a direct role in the biogeochemical carbon cycle by absorbing significant amounts of atmospheric CO_2_, making them important mitigators of climate change due to their carbon storage in soil in quantities greater than other ecosystems [6,7]. Studies have shown destabilization in species diversity in mangrove soils, with an increase in phytopathogenic species in response to climate changes [8,9,10].

*Rhizophora mangle* store large amounts of carbon both in their biomass and sediments, playing a pivotal role in carbon and nutrient retention [11]. The carbon stored in mangrove sediments exceeds that found in most terrestrial soils, making the destruction of these ecosystems a significant threat to the global carbon balance [11,12].

Climate change refers to long-term variations in average temperature and the pattern of extreme weather events. While there are several discussions about its causes, it is widely recognized that global climate change is happening and that human activities play an important role in this process, as these changes have a negative impact on human health [13,14].

In fact, climate pattern changes, driven by global warming, are reshaping the global epidemiological landscape. Changes in variables such as temperature, precipitation, and the frequency of extreme weather events are affecting the dynamics of various infectious diseases, especially those sensitive to climate [15,16].

The current narrative synthesis aims to offer a comprehensive and interpretative perspective by highlighting the diversity of fungi present in mangrove sediments and examining how climate change and anthropogenic pollution affect their composition and functionality. By synthesizing these interactions, this review seeks not only to identify the contributions and limitations in the existing literature but also to provide a robust scientific foundation for mangrove conservation and the development of strategies to mitigate environmental impacts on these critical ecosystems.

**Figure 1:**
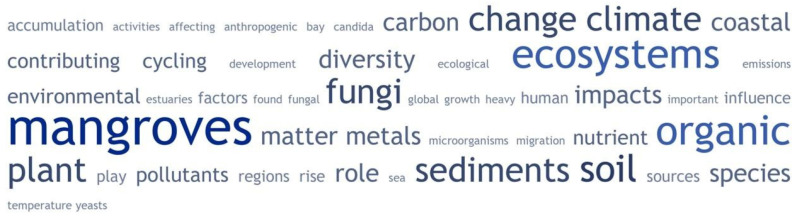
Word cloud based on the 50 most used words in this review.

The 110 selected studies were published across a wide range of years from 1988 to 2024. The studies included in this review were selected based on their relevance to the ecological functions of fungi in mangrove ecosystems and their responses to environmental stressors. Articles were screened for eligibility by reading titles, abstracts, and, where necessary, full texts. Priority was given to publications addressing fungal diversity, biogeochemical cycles, carbon storage, and fungal adaptations to saline and anoxic conditions typical of mangrove ecosystems. Studies related to the effects of pollutants and climate change on fungal communities were also included. Figure 1 presents a word cloud (https://tagcrowd.com/ (accessed on 4 April 2025)) highlighting the 50 most used words in the current review. The color intensity and the size of the words represent, in increasing order, the number of citations used in the text.

## 2. Mangroves: General Characteristics

Mangroves are defined as coastal ecosystems located in tropical and subtropical regions, functioning as a transition zone between marine and terrestrial environments, typically found at the mouths of rivers, estuaries, and coastal lagoons [17]. These ecosystems play a crucial role in ecological balance due to coastal stabilization, contributing to the filtration of sediments and debris through their roots, and providing organic matter that supports the coastal trophic chain. They serve as habitats for characteristic flora and fauna, including both resident and migratory species, while also contributing to nutrient cycling in biogeochemical cycles [18,19].

The organic matter content in mangrove soils can vary; however, these soils are acidic, with a pH ranging from 4.1 to 4.6, like other ecosystems like Atlantic Forest and *restinga*, a Brazilian coastal ecosystem with sandy soils and unique vegetation, part of the Atlantic Forest. The presence of organic carbon in the soil can vary widely, with reported values ranging from 2.9 to 185.6 g/kg in different regions and ecosystems [20,21]. These characteristics influence the composition and activity of microbial communities in soils, affecting their conditions for survival and growth. In addition to soil characteristics, variations in the amount of organic matter (litter) in mangroves may differ, influencing microbial counts among the soils Leff et al. [20].

In a study conducted in the mangrove at the mouth of the Açu River, located in the state of Rio Grande do Norte, the presence of rich vegetation was observed, with a predominance of species such as *Avicennia schaueriana* and *Rhizophora mangle*, which are essential for the carbon sequestration process. The results indicated that these mangroves play an important role in capturing atmospheric carbon, which is stored in the sediment, being crucial for mitigating the impacts of climate change [22,23,24].

Wood et al. [23] mangroves, despite housing only a few tree species, produce large quantities of litter daily, which are responsible for retaining significant amounts of compounds and important nutrient sources returned to the soil [22,23]. The mangrove leaves, especially the decomposing leaves, play an important role in the nutrient cycling within the ecosystem. As the litter decomposes, it releases essential nutrients such as nitrogen, phosphorus, and potassium, which are crucial for sustaining plant growth. The organic matter released by mangrove litter provides a source of nutrients that support microbial communities and higher trophic levels. Staelens et al. [22] in particular, the nitrogen cycle is strongly influenced by the decomposition of the litter, with the process supporting both microbial and plant communities by converting organic nitrogen into forms that are more readily available to plants.

Martins et al. [21] however, much of this litter is carried away by tides, which also influence the high moisture content of the soil. Nutrient cycles are influenced by ocean currents, and the presence of microorganisms can contribute to biomass cycling, allowing for greater sustainability. Therefore, analyzing the influence of microorganisms present in ecosystems such as mangroves is of utmost importance [24].

Mangrove forests are formed by the flooding of coastal areas by tides and the transport of sediments from rivers and oceans. Originating from the regions of the Indian and Pacific Oceans, mangrove species spread around the world through ocean currents during the separation of continents. They occupy areas such as estuaries, coastal lagoons, bays, and deltas, where tides play a crucial role in the transport of sediments and organic matter. The amount of freshwater that mangroves receive is also essential for their development and maintenance [25]. Water circulation causes the mixing of freshwater and saltwater, creating an estuarine environment. This process slows down the transported sediments, allowing them to aggregate through flocculation, forming fine sediments rich in silt, clay, and organic matter, which favor plant growth [11]. The decomposition of leaves and animal remains, along with materials brought by winds, waves, and currents, contributes to substrate formation. The mineral part of the soil consists of products from the decomposition of rocks and volcanic, granitic, or sedimentary materials, mixed with plant and animal remains [24]. Mangroves act as biological filters, contributing to the removal of particles and impurities from water. The action of bacteria, such as sulfate-reducing bacteria, decomposes organic matter using sulfate from seawater, creating an anaerobic environment [26]. Recent studies conducted in the mangroves of Baixada Santista (state of Sao Paulo, Brazil) demonstrated that, despite anthropogenic pollution caused by the accumulation of heavy metals like manganese, lead, and cadmium, plants adapt to the hydric balance, limiting the entry of metals and salts [26].

The Baixada Santista is a region with a relief characterized by vast alluvial plains, estuaries, and mangroves, whose geological dynamics directly affect ecological processes and land use [26]. The local plains and lowlands are strongly influenced by the hydrological behavior, such as the alternation between dry and rainy periods, and by the action of tides and floods, which contribute to erosion and sediment deposition [26].

This process shapes the lowland and plain areas, with temporary flooding during the rainy season. The interaction between the relief and the tides facilitates the formation of estuaries and mangroves, which are essential ecosystems for local biodiversity. These ecosystems protect coastal areas against erosion, serve as nurseries for various marine species, and play a vital role in the region’s nutrient cycle [26].

Microorganisms are essential for the sustainability of ecosystems, playing a crucial role in the decomposition of organic matter and mineralization [26]. The presence of microorganisms, such as bacteria, fungi, and actinobacteria, varies significantly between ecosystems; however, their populations and activities can be influenced by changes in the physical and chemical characteristics of the soil, such as organic matter content and pH, as well as seasonal variations and maritime influences [12]. Nevertheless, the oxygen availability in the soil can be low due to the influence of flooded regions, affecting the microbial composition [27].

However, mangroves are recognized for their ecological benefits and environmental importance, such as the contribution of terrestrial organic matter to the oceans and their high biodiversity [28,29]. Nonetheless, their conservation has rapidly deteriorated due to industrial development, local human activities, pollution, droughts, erosion, sedimentation, and variations in water salinity. These factors cause stress to the trees, making them vulnerable to opportunistic pathogens and diseases, which exacerbates the degradation of mangroves.

### 2.1. Diversity of Fungi in Mangrove Sediments

Fungal diversity can vary depending on the studied ecosystem. A study conducted in the mangroves of southern China found that the frequency of fungi and actinobacteria was lower compared to bacteria. In contrast, in the mangroves of Tamil Nadu, India, the *counts* of actinobacteria were much higher compared to those in mangrove and restinga soils [19].

In Brazilian mangroves of Duque de Caxias, Jequiá, and Restinga de Marambaia (state of Rio de Janeiro), the most dominant fungi belonged to the genera *Aspergillus* and *Penicillium* [30,31]. In the Atlantic Forest soil, the counts were like those from the mangroves of Manakkudi, India [19,29]. The frequency of fungi was particularly low in mangrove soil compared to the Atlantic Forest and other mangroves, such as that of Suva, Fiji. Although microorganisms, especially bacteria and fungi, account for 91% of the total biomass of these ecosystems, the fungal fraction remains poorly studied Andreote et al. [19,29]. These results demonstrate that the growth of fungi in amphibious regions, such as mangroves, can be limited by factors that influence their growth, such as high moisture concentration, soil chemical composition, and flooding of areas with brackish water from the sea [30,31,32].

In sediment and water samples from Guanabara Bay and Sepetiba Bay, located in the state of Rio de Janeiro, 47 yeast species were identified, with 34 belonging to *Candida*, with *C. tropicalis* being the most frequent. Opportunistic pathogenic species found included *Nakaseomyces glabratus* (formerly *Candida glabrata*) [33], *C. guilliermondii*, *C. parapsilosis*, and *C. krusei*. The research also documented the presence of other yeasts such as *Rhodotorula* spp., *Cryptococcus* spp., *Trichosporon* spp., *Kluyveromyces aestuarii*, and *Geotrichum* spp. [31].

These yeasts thrive in tropical estuarine environments, adapted to fluctuating environmental conditions, such as salinity and sediment composition. The distribution of yeasts in Guanabara Bay and Sepetiba Bay showed seasonal variations, with a higher abundance of yeasts during the rainy season, when the amount of organic matter and nutrients in the water increases. Additionally, the research observed that the isolated yeasts exhibited resistance to high concentrations of salt, which is a key characteristic for survival in these coastal ecosystems. The diversity of yeasts in the mangroves of both bays was influenced by factors such as salinity, substrate type, and the anoxic conditions of the environment. The yeasts demonstrated a high capacity for adaptation to these extreme conditions, highlighting the importance of these fungi in the stabilization and ecological balance of mangrove ecosystems. Yeasts also play essential roles in the nutrient cycle, breaking down organic matter and contributing to nutrient dynamics in the soil and water [31,32,33]. The distribution of yeasts in Guanabara Bay and Sepetiba Bay showed seasonal variations, with a higher abundance of yeasts during the rainy season, when the amount of organic matter and nutrients in the water increases. Moreover, the research observed that the isolated yeasts exhibited resistance to high concentrations of salt, which is a fundamental characteristic for survival in these coastal ecosystems [31].

The diversity of yeasts varies according to local ecological factors, such as hydrodynamic dynamics and seasonal variations, and they have potential for biotechnological applications, such as biodegradation and bioremediation. A 2013 study also identified filamentous fungi like *Fusarium* spp., *Cladosporium* spp., and *Penicillium* spp. in the sediments of the Guanabara Bay mangrove [31].

### 2.2. Role of Fungi in Decomposing Organic Matter and Nutrient Cycling

Mangroves play a crucial role in climate regulation by acting in carbon sequestration, drawing carbon from the atmosphere through biogeochemical cycles and retaining it in the soil [34]. Along with tropical forests, mangroves are among the most effective ecosystems in combating global warming due to their capacity for carbon retention during photosynthesis [24,35]. They are vital in retaining carbon and nutrients, accumulating more sediments in terrestrial soils than other ecosystems [35]. Carbon, in the form of CO_2_, is involved in various natural processes such as photosynthesis, respiration, oceanic dissolution, and the decomposition of organic matter [36].

In the context of atmospheric carbon sequestration, the symbiotic relationship between mycorrhizal fungi and plants enhances the use of the CO_2_ reservoir, benefiting the development of both organisms. Moreover, they play an important role in the transport, storage, and release of nutrients such as carbon, phosphorus, and nitrogen [37].

Fungi belonging to Basidiomycota and Ascomycota play an essential role in ecosystems by contributing to nutrient cycling, carbon storage, and decomposition. They also engage in mutualistic symbiotic relationships, which help sustain the survival of living organisms [37]. These contributions to ecosystem conservation must be recognized and integrated into conservation policy and the evaluation of the ecosystem services provided by forests [37].

Ectomycorrhizae, fungi from the Basidiomycota and Ascomycota phyla, grow externally to plant roots, excreting extracellular enzymes in the soil to metabolize organic matter [38,39]. The availability of organic matter in the soil is influenced by two factors: the growth rate and the nutritional status of the plants [40]. The environmental heterogeneity of ectomycorrhizae is a pattern of similarity between samples collected from the soil surface and deeper layers, influenced by nutrient availability and the extent of root colonization [41].

Ectomycorrhizae are associations between fungi, typically from the Basidiomycota and Ascomycota phyla, and plant roots. In these associations, the fungus grows externally to the plant’s roots, forming a mycelial layer around the root and penetrating the intercellular space of the root cortex [1,42].

Ectomycorrhizae play a vital role in the absorption of nutrients such as phosphorus, nitrogen, and other essential elements for plant growth. Additionally, these fungi enhance the plant’s resistance to adverse environmental conditions such as drought, high salinity, and heavy metal toxicity [1]. While not extensively documented in mangrove ecosystems specifically, ectomycorrhizae could potentially be important for plants like Rhizophora mangle (red mangrove) that face conditions of high salinity and soils with a low nutrient availability. If present in mangroves, these fungal associations might help these plants adapt to extreme conditions by improving nutrient absorption and resistance to environmental stressors. Moreover, the symbiotic interaction between the fungus and the plant results in a mutual exchange of benefits: the fungus receives carbohydrates and other organic compounds from the plant, while the plant obtains minerals and other essential nutrients from the fungus [42].

Endomycorrhizae, fungi from the Glomeromycota phyla, exhibit slower internal growth within plants, colonizing their roots and thriving in environments rich in sugars [39,42]. These fungi form structures called arbuscules, which are sites for nutrient exchange between the fungus and the plant. Endomycorrhizae are particularly common in vascular plants and are responsible for increasing the plants’ ability to absorb water and nutrients, such as phosphorus, from the soil [42]. These fungi are most common in vascular plants, representing approximately 80% of the intracellular distribution in plant root cortical cells [42,43]. Although the research on endomycorrhizae in mangrove environments is limited, these fungi could theoretically play a role in the adaptation of plants to water-saturated environments and nutrient-poor soils. In terrestrial ecosystems, endomycorrhizae aid in the absorption of phosphorus and other minerals, which is essential for plant growth, especially in low-fertility soils. Similar benefits might occur in mangroves if these associations are confirmed. Moreover, these symbiotic associations contribute to the plants’ resistance to diseases, water stress, and other adverse environmental factors [42].

The mycorrhizal symbiosis also increases plant tolerance to environmental stressors such as acidity, heavy metal toxicity, high soil temperatures, and diseases affecting the plant’s vascular system [44]. Additional benefits include erosion control and improved soil aggregation through the extramatricial mycelium [45]. The diversity of mycorrhizae in a given habitat is influenced by factors such as plant community age, and the chemical, physical, and biological properties of the soil and climate [46].

The ecological interactions of fungi are primarily characterized by a symbiotic relationship between plants and mycorrhizae, which contribute to the absorption of minerals and water from the soil [47,48]. This association arises due to carbon deficiency, which is a crucial source for the metabolism of microbial species [49]. Symbiotic relationships are defined as a mutually beneficial interaction between two organisms [49].

Mycorrhizae were first described in 1885 by German botanist Albert Bernard Frank, referring to the organic union of fungal mycelium and plant roots, resulting in a symbiotic relationship [50]. These relationships are essential for nutrient cycling, supporting plant growth, resilience to environmental stressors, and carbon sequestration [51]. During this process, both organisms benefit: the fungus receives nutrients like sugars, vitamins, and lipids from the plant, while the plant obtains essential minerals and nutrients from the fungus Junior et al. [52].

Local development, driven by industrial growth, has caused negative impacts due to the unregulated occupation of land, generating environmental impacts on ecosystems such as mangroves. In recent years, these ecosystems have had their territorial extents compromised due to erosion, river siltation, the deterioration of water quality, and the loss of biodiversity [2]. Numerous consequences caused by anthropogenic practices have led to increased greenhouse gas emissions and reduced species diversity [53].

It is important to note that, while the mycorrhizal associations described above are well-documented in terrestrial ecosystems, their presence, extent, and ecological significance in mangrove ecosystems specifically remain largely hypothetical and require further investigation. The unique conditions of mangrove environments, including regular tidal flooding with saline water, may result in different fungal associations than those observed in typical terrestrial systems. Future research should focus on confirming the presence and understanding the specific roles of mycorrhizal fungi in mangrove ecosystems.

### 2.3. Impacts of Human-Induced Climate Change

Human-induced climate change results from over a century of net greenhouse gas emissions related to energy use, land use changes, and consumption and production patterns.

The top 10% of households with the highest per capita emissions account for 34–45% of global domestic emissions, while the middle 40% contribute 40–53%, and the bottom 50% account for 13–15% [54]. Additionally, the share of urban emissions increased from about 62% to 67–72% of the global total between 2015 and 2020 [54]. Factors contributing to urban emissions are complex, including population size, income, level of urbanization, and city layout [55].

Climate change has gradually increased sea levels and the intensity of external climate events, such as storm surges, which can submerge, erode, and threaten coastal environments like mangroves, due to the loss of mass from glaciers and ice. Furthermore, biogeochemical cycles will be affected by the intensification and increase in CO_2_ in the atmosphere and ocean acidification [55,56]. The biogeochemical filtering and transformation of substances may be impacted by rising sea levels in extensive areas of mangrove territory [57].

Locally, the distribution and composition of mangroves remain nearly unchanged when the rates of sea level rise and sediment accumulation are roughly equivalent. However, when the rate of sea level rise exceeds the sediment accumulation rate, mangroves may be lost as the average high tide exceeds substrate elevation. This effect will be more pronounced on low-lying limestone islands with a negligible allochthonous sediment input, such as some Caribbean islands [58], and in deltas of several major tropical rivers where subsidence and the disappearance of many deltaic islands are underway, such as in the Sundarbans, a mangrove area in the Bay of Bengal, India [11,25,57].

Considering all these climate changes, mangroves depend on the availability of areas to which they can migrate in search of more favorable conditions, as their territories are geographically reshaped due to rising tides [56]. These changes affect the biodiversity of animals and plants and also impact the metabolic activity and symbiotic relationships of microorganisms, including fungi, which can be pathogens to plants, animals, and humans. As these negative changes intensify, there is an increased risk of emerging diseases caused by these organisms [59,60]. Since 1950, the rise in global temperature has been causing direct and indirect impacts on humans and terrestrial ecosystems, with the risk worsening due to the gradual increase in temperature [55]. This increase is caused by anthropogenic emissions of greenhouse gases; however, according to the United Nations, climate change refers to long-term changes in temperature and climate patterns. These climate changes have generated negative impacts on biological, agricultural, and human systems, representing risks for various sectors, including the economic, health, and environmental sectors [55].

In recent decades, the causes, impacts, and risks of climate change, as well as mitigation and adaptation measures, have been widely studied. The Intergovernmental Panel on Climate Change (IPCC) plays a central role in assessing global scientific research on climate change, analyzing trends, sources, impacts, and options for combatting it [55].

The IPCC highlights that the last five years have been the hottest on record, with extreme weather events such as heatwaves, floods, and severe droughts becoming more frequent and intense. These events have caused substantial damage to the infrastructure, ecosystems, and vulnerable populations, particularly in developing countries. According to the IPCC (2023), climate change is already a reality experienced in various parts of the world. Evidence of global temperature rise, sea level rise, changes in precipitation patterns, and the intensification of extreme weather events are becoming increasingly evident [55]. The continuous increase in global temperature, which has already surpassed 1.1 °C since the pre-industrial era, is directly linked to human activities, especially the emission of greenhouse gases (GHGs) from fossil fuel combustion, deforestation, and unsustainable agricultural practices. Global ecosystems are being profoundly impacted by climate change. The increase in temperatures and changes in precipitation patterns have significantly affected biodiversity [55].

The IPCC (2023) points out that mangroves, coral reefs, and tropical forests are among the most vulnerable ecosystems, as their ability to adapt to climate change is limited. The loss of these ecosystems would affect the protection against natural disasters, food security, and the health of marine and terrestrial ecosystems.

The IPCC uses a combination of observations, assumptions, and future models for its assessments, assigning probabilities to climate changes based on the policies and actions of countries. In addition to the IPCC studies, various international research efforts have been conducted to understand and mitigate climate changes, with governments and institutions collaborating in international agreements to address the problem [55].

### 2.4. The Effect of Climate Change on the Proliferation of Fungal Diseases

Global climate change is altering patterns of infectious diseases around the world. While the seasonality of diseases related to gastrointestinal and respiratory infections is well-understood, the impact of climate on fungal diseases remains complex and poorly comprehended [61].

The relationship between climate change and infectious diseases has attracted increasing attention in recent decades. Among the wide range of diseases affected by climate change, fungal infections stand out due to their growing prevalence and the changing environmental conditions that support their spread. As global temperatures rise and precipitation patterns shift, the conditions for fungal pathogens to thrive have expanded. The implications of these changes for human health, particularly in the context of invasive mycoses, have raised significant concerns among researchers and public health professionals [60].

The worsening of global warming is contributing to the rise in fungal-related diseases, as thermotolerant fungi possess pathogenic potential due to their ability to survive at mammalian temperatures. This fact makes fungi a potential global health threat [61].

Fungi can benefit from natural selection, where species that adapt better to high temperatures through thermal selection—an important barrier that prevents the development of infections by most fungi—can thrive [36,61]. Oliveira et al. [62] conducted a study showing that some phytopathogenic fungi respond to a temperature increase of 2 °C and reduced soil water availability, contributing to the spread of soil-borne diseases.

Mangroves are impacted by anthropogenic activities, such as oil spills, which affect the diversity present in this ecosystem [63]. Anthropogenic pollution in this ecosystem acts as a selective pressure, affecting the diversity of fungal species richness due to influential variables [63]. Many microorganisms are capable of degrading polycyclic aromatic hydrocarbon (PAH) or domestic sewage [64].

The discharge of domestic sewage leads to a high influx of nutrients from organic and inorganic compounds, contributing to the increased occurrence of invasive pathogen development [65].The disposal of domestic sewage contributes to the accumulation of waste in the soil, leading to environmental degradation in the area [26,66,67].

Worldwide, mangroves have decreased by 35% to 86%, depending on the region, in response to direct human pressures, primarily associated with urban expansion and aquaculture [62]. The main determining factor for the resilience of mangroves to rising sea levels and warmer conditions related to climate change is migration inland and toward the poles. Evidence of poleward migration suggests a predominant influence from the decreased frequency of extreme cold events.

However, the additional expansion and the survival of the ecosystem as a whole are primarily determined by local and regional factors. The local arid climate appears to be a limiting factor for mangroves on the Pacific coast of South America, for example. The coastal topography and/or sediment addition that allows the system to migrate inland and maintain adequate surface elevation are also involved in the poleward migration of mangroves. Consequently, coastal development, such as urban expansion and rigid coastal engineering works, becomes even more significant in this context [63].

Eslami et. al. [68] migration inland and changes in the covered area seem to be the primary response of mangrove ecosystems to climate change and depend on various other factors that determine the environment, including precipitation and temperature variability [68,69,70]. The ability of fungi to adapt to higher temperatures and altered environmental conditions makes them an increasing concern for public health. Addressing the impact of climate change on fungal diseases requires a multifaceted approach, including better surveillance of fungal pathogens, investments in healthcare infrastructure, and more research on the relationship between climate and the emergence of fungal diseases [60].

The geomorphology of the coastal plain and sedimentation rates that accompany the rate of sea level rise are key parameters controlling the magnitude of inland migration. In environments with a sufficient allochthonous sediment supply and/or organic matter production and accumulation, and a suitable gradient of terrestrial surface elevation, rising sea levels do not pose a threat to mangroves. This is generally true for river-dominated environments, tidal areas with an abundant sediment supply, and coastlines experiencing progradation [69]. Krauss, et al. [71] however, land elevation or subsidence, groundwater influx, plant and soil processes, and whether the coast is accreting or eroding also play important roles in determining the extent of inland migration [71,72,73].

Depending on sediment supply, mangroves accumulate peat or mud, allowing them to adjust to rising sea levels. Existing data show that sedimentation rates often exceed current rates of sea level rise, facilitating inland migration [57,73].

### 2.5. Chemical Pollutants in Mangroves

Kulkarni et al. [74], mangroves are ecosystems exposed to pollution from the sea (e.g., microplastics) and the mainland, for example, due to the dumping of industrial effluents and wastewater, which may contain toxic metals, pharmaceuticals, industrial contaminants, and pesticides [74,75,76]. Inorganic pollution, including major and trace elements, result from human activities such as industry, maritime traffic, fertilizer use, and sewage, as well as from oil spills, navigation, and mining. Gustin et al. [77], the accumulation of these elements and organic contaminants (e.g., persistent and pseudo-persistent organic pollutants) in mangrove ecosystems can affect the biodiversity of the food chain, including fish, seabirds, and humans consuming food from these ecosystems. Additionally, some toxic elements and organic compounds can negatively affect human health through the consumption of seafood, including carcinogenic effects and damage to the nervous and renal systems [77,78].

The presence of mercury (Hg) was identified in a mangrove area in the Baixada Santista (state of São Paulo). Mercury participates in the biogeochemical cycle of the atmosphere, but it can be found in high quantities in sediment and in the biota (mainly at the top of the chain) of some rivers, lakes, and reservoirs [62]. Obrist et al. [78] mercury is found in mangrove sediments from continental, marine, and atmospheric sources, and can be associated with the organic phase due to the limited mobility [79,80]. In addition, mangrove vegetation plays a significant role in Hg deposition due to root absorption [77,78,80]. The most toxic form of Hg, methylmercury (MeHg), can be absorbed by humans up to 90% through the consumption of contaminated food, especially fish, which can biomagnify 1000 to 10,000 times more than water. MeHg exhibits toxicity by binding to proteins and inhibiting enzymatic activities, driven by its high affinity for sulfur atoms within sulfhydryl groups in the cellular protoplasm. In addition to its high toxicity, MeHg can biomagnify in the food chain, resulting in elevated Hg levels in organisms at the top of the chain [79,81,82].

The Mandovi estuary in India showed higher levels of metals, such as Iron (Fe), manganese (Mn), cobalt (Co), copper (Cu), zinc (Zn), and lead (Pb), from anthropogenic sources like iron ore and petroleum activities in the estuary area [83]. The presence of Cu and Zn in the river mouth region, associated with high levels of organic carbon, indicated organic waste from municipal sewage entering the ecosystem [49]. Carcinogenic metals, such as hexavalent chromium (Cr^6+^) and pentavalent arsenic, have been identified as inorganic pollutants in the environment and act as contaminant sources in mangrove sediments [84].

Some metals dissolved in the environment can bioaccumulate in organisms at the base of the aquatic food chain, such as phytoplankton and zooplankton [85]. Dehghani et al. [86] these metals can also be absorbed by fish gills, amphipod cuticles, and other organisms. Bioaccumulation in the food chain puts organisms at all levels at risk, from the microscopic base to fish and humans who rely on these organisms as a food source [87,88].

Some metals such as Pb, Cu, Zn, and nickel (Ni) pose a significant threat to the health of aquatic organisms and humans. These metals can cause malformations, genetic anomalies, and cellular changes due to their high neurotoxic potential, with lead being especially harmful to the nervous system. The reference concentration for Pb^2+^ is 0.01 mg.dm^−3^, which is essential for controlling environmental impacts. In the mangroves of Santos, SP, copper and zinc have reference values of 2 mg.dm^−3^ and 5 mg.dm^−3^, respectively, while nickel has a reference concentration of 0.02 mg.dm^−3^. This underscores the need for the continuous monitoring of these metals, as estuarine ecosystems are vulnerable to contamination, affecting aquatic fauna and human health [89,90].

A study in China, in the Pearl and Jiulong River estuaries, showed higher concentrations of Cu, Zn, cadmium (Cd), and Pb in mangrove sediments in Guangdong, Fujian, and Hong Kong compared to other regions like Guangxi and Hainan. Additionally, polycyclic aromatic hydrocarbon (PAH) was found in the mangrove sediments of Hong Kong, Fujian, and Guangdong. Pb, Cd, and mercury (Hg) were also present in mollusks [91,92].

In the Environmental Preservation Area of the São Gonçalo mangroves (Itaoca Environmental Preservation Area, state of Rio de Janeiro, Brazil), which received domestic waste for 28 years, PAH concentrations were found in sediments, such as acenaphthylene and dibenz[a,h]anthracene (DBA). The persistence of pollutants in these environments underscores the need for ongoing monitoring and impact studies on both ecological and human health [92].

Mangroves face a significant load of pollutants from multiple sources, and land-use changes along with increased urbanization in coastal areas exacerbate these impacts. However, stress caused by heavy metals has been widely studied due to economic growth driven by industrialization and urbanization, which produce adverse effects from the accumulation of these elements in mangrove plants. Recent studies show that metal distribution occurs primarily through accumulation in the root level, with limited translocation to the aerial parts of the plant. Nevertheless, roots exhibit a bioconcentration factor for metals such as Cu, Zn, Cd, chromium (Cr), and Hg, while leaves show lower concentrations, indicating the translocation to other parts of the plant [93].

The presence of microplastics (MPs), resulting from increasing urbanization, industrial activities, and inadequate waste management, has become a growing issue in the mangroves of the Americas. A study conducted in a mangrove in Todos os Santos Bay (state of Bahia, Brazil) found 10,782 items.kg^−1^ of MP [94]. Microplastics have emerged as widely distributed contaminants in various ecosystems, with significant implications for the environment and the health of marine organisms [95]. Primary MPs are plastic particles manufactured in microscopic dimensions, released directly into the environment during the production and use of products such as cosmetics, toothpaste, exfoliants, and cleaning products. Due to their small size and widespread use, these MPs are often discarded into the environment, contributing to the contamination of ecosystems, particularly aquatic ones [95]. Secondary MPs are formed by the fragmentation of larger plastics, such as bottles, packaging, and fishing nets, exposed to environmental factors such as photodegradation, wear caused by sea waves and wind, and biological actions. This continuous degradation process results in smaller particles that disperse further into ecosystems over time, intensifying environmental impacts [95]. Microplastics have been found in various environments, including freshwater, estuaries, oceans, beaches, mangroves, and the atmosphere. The spread of MPs is a direct consequence of the extensive use of plastics in modern society and inadequate waste management practices, leading to the continuous introduction of these pollutants into the environment [96].

In Singapore, the presence of emerging pollutants such as bisphenol A (BPA), atrazine, and pharmaceutically active compounds (PhACs), common in the pharmaceutical and agrochemical industries, was observed. The presence of these contaminants in marine ecosystems can pose risks to biodiversity and the health of marine organisms, affecting both lower trophic level organisms and predators. The study highlights the importance of investigating PhAC contamination in mangroves, given their role in the bioaccumulation of these compounds in marine organisms and the potential long-term ecological impacts [97].

Sediment and water samples from the Gaoqiao Mangrove in China revealed the presence of antibiotics such as sulfonamides, fluoroquinolones, and oxytetracycline, with concentrations ranging from 0.15 to 198 ng.L^−1^ in water and from 0.08 to 849 μg.kg^−1^ in sediments. Despite these concentrations, the study observed that, to some extent, mangrove vegetation is able to mitigate antibiotic pollution [98]. Antibiotic pollution in aquatic environments is an increasing concern, particularly due to the rising use of pharmaceutical substances, such as fluoroquinolones, in aquaculture. These compounds have been detected in various coastal zones and marine ecosystems, including mangroves, which are critical ecosystems for biodiversity and coastal protection. Fluoroquinolones, such as norfloxacin, ciprofloxacin, and enrofloxacin, are widely used to treat infectious diseases in aquatic animals, leading to the risk of their residues being released into the environment. The accumulation of fluoroquinolones in the roots of mangrove plants, as observed for NOR, may have implications for bioaccumulation and long-term effects on ecosystem health [99,100,101].

Mangrove plants, to prevent damage to cellular structures and metabolism, have developed resistance to stress generated by human development processes. This is achieved through the production of antioxidant mechanisms, both enzymatic and non-enzymatic, that neutralize reactive oxygen species (ROS). This phenomenon indicates that the plant is activating its defense system, increasing enzyme activity in parallel with concentrations of toxic metals to cope with the stress [92].

### 2.6. The Role of Plants, Microbes, and Fungi in Remediation

Heavy metal contamination has become one of the most pressing environmental issues, as metals such as cadmium (Cd), nickel (Ni), lead (Pb), and mercury (Hg) pose significant risks to human health, aquatic life, and terrestrial ecosystems [102]. Munk et al. [102].the process of bioremediation, which involves the use of living organisms to degrade or detoxify environmental pollutants, has gained significant attention as a sustainable alternative to conventional pollution control methods.

Traditional methods for removing heavy metals from contaminated environments, such as chemical precipitation, electrochemical treatment, and adsorption, are often costly and environmentally invasive. As a result, bioremediation, especially through the use of fungi, has emerged as a more economical and eco-friendly solution. A study conducted in 2019 explored the potential of *Phanerochaete chrysosporium* for the bioremediation of two common heavy metals, cadmium (Cd) and nickel (Ni), from contaminated environments [102]. The study focused on the ability of *Phanerochaete chrysosporium* to remove these metals from both liquid and solid substrates under laboratory conditions.

The results showed that *Phanerochaete chrysosporium* was effective in removing both cadmium and nickel, with greater efficiency observed in cadmium removal [103] the fungus exhibited a high capacity for cadmium uptake, with the substantial removal of the metal occurring within the first 72 h of exposure. Biosorption and bioaccumulation mechanisms were identified as the primary pathways for cadmium removal, with the metal predominantly localized in the fungal biomass [102,104].

Scientific studies have utilized white rot fungi (WRF), a group of organisms known for their remarkable ability to degrade complex compounds in the environment. These fungi possess significant biodegradability capabilities, making them promising candidates for various environmental applications. WRF can degrade and transform macromolecules like lignin and xenobiotics through synergistic mechanisms involving enzymatic systems and free radicals. Due to their versatility and broad substrate range, as well as the presence of ecologically friendly degradative enzymes, WRF have been widely applied in the remediation of organic pollutants such as active pharmaceutical compounds [105], polycyclic aromatic hydrocarbon (PAH) [106], endocrine-disrupting compounds (EDCs) [103], herbicides Kaur et al. [103], and pesticides [107].

In recent decades, the application of WRF in environmental remediation has grown significantly, as knowledge about these organisms expands, and advances are made in biotechnology for heavy metal pollution remediation. The results are impressive, as in the case of *Phanerochaete chrysosporium*, which, when modified with polyethyleneimine (PEI), shows higher efficiency in removing Cr^6+^ from wastewater. Under optimal conditions, this fungus can remove approximately 344.48 mg/g of Cr^6+^, with about 32.5% of this amount converted into trivalent chromium (Cr^3+^) [108] *phanerochaete chrysosporium* also excels in removing lead (Pb) through extracellular adsorption and intracellular accumulation, achieving a maximum removal efficiency of 91.3% when the Pb concentration is 50 mg/L [109].

Additionally, the immobilization of *Agaricus bitorquis* using alginate beads has shown promising results, being capable of remediating up to 205.1 mg/g of heavy metals [108]. *Phanerochaete chrysosporium* is also effective in the remediation of cadmium (Cd) and nickel (Ni), with adsorption efficiencies of 96.23% and 89.48%, respectively, and adsorption capacities of 71.43 mg/g for Cd and 46.50 mg/g for Ni. Other fungi, such as *Schizophyllum commune* and *Pleurotus ostreatus*, also demonstrate high removal capacities for uranium, with removal rates of up to 463.2 ± 38.1 μmol/g and 441.8 ± 79.4 μmol/g, respectively. It is important to note that, while *Phanerochaete chrysosporium* and other white rot fungi have demonstrated significant bioremediation potential in laboratory studies and various terrestrial ecosystems, their natural occurrence in mangrove ecosystems has not been extensively documented. The examples presented above illustrate the general bioremediation capabilities of fungi that could potentially be applied to mangrove restoration efforts, rather than describing fungi that are known to be native to mangrove ecosystems.

Mangrove-associated fungi with bioremediation potential do exist, including species from genera such as *Aspergillus, Penicillium, and Trichoderma*, which have been isolated from mangrove sediments and shown to tolerate and/or accumulate heavy metals [30,33]. However, the application of non-native fungi with proven bioremediation capabilities, such as *P. chrysosporium*, represents a potential biotechnological approach that would require a careful ecological assessment before implementation in mangrove ecosystems.

The advancement of this field would benefit from identifying and characterizing indigenous mangrove fungi with natural bioremediation capabilities, while also exploring the potential for controlled applications of efficient bioremediation fungi in contaminated mangrove sites. Additionally, developing fungal-based bioremediation technologies specifically adapted to the unique conditions of mangrove ecosystems, including salinity fluctuations, periodic inundation, and anoxic sediments, could provide valuable tools for restoration efforts in these critical coastal environments.

As research in fungal bioremediation continues to advance, the role of fungi in the sustainable management of polluted ecosystems is expected to expand, offering innovative solutions for reducing environmental contamination and promoting ecological restoration.

## 3. Conclusions

This review highlighted the crucial role of fungi in processes such as organic matter decomposition and the carbon cycle, particularly in complex ecosystems like mangroves. Despite significant advances in understanding fungal diversity, many aspects remain insufficiently explored, especially in the context of environmental changes and contamination. Mangroves, with their unique bioconcentration mechanisms and antioxidant systems, demonstrate remarkable resilience to high pollution levels, including heavy metals.

In this context, fungal bioremediation has emerged as a promising approach to addressing environmental contamination in coastal areas. Fungi possess unique capabilities to degrade or detoxify pollutants, including heavy metals, through mechanisms such as biosorption and bioaccumulation. These abilities position fungi as valuable allies in mitigating pollution and restoring ecosystems, particularly in mangrove habitats.

Future research should focus on understanding how climate change, rising temperatures, and pollution influence fungal diversity and functionality in mangroves. Beyond bioremediation applications, several promising research directions could significantly advance our understanding of mangrove fungal communities. These include comprehensive metagenomic and metatranscriptomic analyses to fully characterize the taxonomic and functional diversity of fungi across different mangrove zones and geographical regions; an investigation of fungal–plant symbioses specific to mangrove ecosystems, particularly potential mycorrhizal or endophytic relationships that may enhance plant resilience to environmental stressors; an exploration of fungal succession patterns during mangrove development and restoration; a characterization of novel secondary metabolites from mangrove fungi with potential pharmaceutical or industrial applications; and the development of fungal indicators for a mangrove ecosystem health assessment. Additionally, understanding the complex interactions between fungi and other microbial communities in mangrove sediments could provide insights into biogeochemical cycling and ecosystem functioning.

Longitudinal studies are essential for tracking changes in fungal communities over time, as well as exploring their interactions with other microbiota. However, challenges remain, including the need for more comprehensive data on fungal species distribution and abundance, which can be addressed through advanced methodologies. A greater integration between ecological studies and research on heavy metal contamination, as well as investigations into the impact of invasive fungal species, will be crucial for advancing our understanding of these ecosystems and their conservation.

## References

[1] Pupin B., Nahas E. (2014). Microbial populations and activities of mangrove, restinga and Atlantic forest soils from Cardoso Island, Brazil. J. Appl. Microbiol..

[2] Alves J.R.P. (2001). Manguezais: Educar para Proteger.

[3] Rodrigues S.A. (1995). O Manguezal e Sua Fauna.

[4] Ghizelini A.M., Macrae A. (2013). Orient. Diversidade e Potencial Biotecnológico de Fungos Isolados de Sedimentos de Manguezais do Rio de Janeiro, Brasil. Ph.D. Thesis.

[5] Ghosh A., Dey N., Bera A., Tiwari A.B., Sathyaniranjan K.B., Chakrabarti K., Chattopadhyay D. (2010). Culture independent molecular analysis of bacterial communities in the mangrove sediment of Sundarban, India. Saline Syst..

[6] Kauffman J.B., Bernardino A.F., Ferreira T.O., Giovannoni L.R., Gomes L.E., Romero D.J., Jimenez L.C.Z., Ruiz F. (2018). Carbon stocks of mangroves and salt marshes of the Amazon region, Brazil. Biol. Lett..

[7] Alongi D.M. (2008). Mangrove forests: Resilience, protection from tsunamis and response to global climate change. Estuar. Coast. Shelf Sci..

[8] Ghizelini A.M., Martins K.G., Gießelmann U.C., Santoro E., Pasqualette L., Mendonça-Hagler L.C.S., Rosado A.S., Macrae A. (2019). Fungal communities in oil contaminated mangrove sediments—Who is in the mud?. Mar. Pollut. Bull..

[9] de Oliveira L.A., Segundo W.O.P.F., de Souza E.S., Peres E.G., Koolen H.H., de Souza J.V. (2022). Ascomycota as a source of natural colorants. Braz. J. Microbiol..

[10] Dufossé L., Fouillaud M., Caro Y., Mapari S.A.S., Sutthiwong N. (2014). Filamentous fungi are large-scale producers of pigments and colorants for the food industry. Curr. Opin. Biotechnol..

[11] Alongi D.M. (2002). Present state and future of the world’s mangrove forests. Environ. Conserv..

[12] Lovelock C.E., Ellison J. (2007). Vulnerability of mangroves and tidal wetlands of the Great Barrier Reef to climate change. Climate Change and The Great Barrier Reef: A Vulnerability Assessment.

[13] Haines A., Kovats R.S., Campbell-Lendrum D., Corvalan C. (2006). Climate change and human health: Impacts, vulnerability, and mitigation. Lancet.

[14] Yang J., Gong P., Fu R., Zhang M.H., Chen J.M., Liang S.L., Xu B., Shi J.C., Dickinson R. (2013). The role of satellite remote sensing in climate change studies. Nat. Clim. Change.

[15] Panic M., Ford J. (2013). Climate change and human health: Impacts and adaptation strategies. Glob. Health Action.

[16] Hoeg L. (2019). Climate change and the risk of infectious diseases: A global health perspective. Environ. Health Perspect..

[17] Rengasamy A. (2006). Distribution and seasonal variation of trace metals in surface sediments of the Mandovi estuary, west coast of India. Estuar. Coast. Shelf Sci..

[18] Thatoi H., Behera B.C., Mishra R.R., Dutta S.K. (2013). Biodiversity and biotechnological potential of microorganisms from mangrove ecosystems: A review. Ann. Microbiol..

[19] Andreote F.D. (2012). Microbial diversity and ecosystem functions in mangrove forests. Sci. Total Environ..

[20] Leff J.W., Jones S.E., Prober S.M., Barbera P., Bates S.T., Borer E.T., Firn J., Harpole W.S., Hobbie S.E., Hofmockel K.S. (2011). Variation in organic carbon concentrations in soils across different ecosystems. Soil Biol. Biochem..

[21] Martins L.F., Silva R.F., Oliveira A.P., Santos J.C., Almeida M.T., Costa P.R., Pereira L.A., Rodrigues F.J., Souza D.M., Lima E.R. (2008). Soil organic carbon in agricultural and forest ecosystems: A comparative study. Geoderma.

[22] Staelens J., De Schrijver A., Verheyen K., Verhoest N.E.C., Boeckx P., Nachtergaele L., Luyssaert S., Van den Berge J., Van den Bulcke J., Muys B. (2011). The role of mangrove litter in nutrient cycling and organic matter retention. Wetl. Ecol. Manag..

[23] Wood A.R., Smith T.J., Anderson G.H., Brown M.B., Carter R.L., Davis J.P., Evans K.L., Foster J.R., Green P.A., Hall R.J. (2012). Carbon sequestration in mangrove forests: The contribution of litter production to soil organic carbon storage. Glob. Biogeochem. Cycles.

[24] Amaro V.E., Rocha-Junior J.M. (2012). Avaliação ecológico-econômica do manguezal na foz do rio Açu/RN: O sequestro de carbono e a importância da aplicação de práticas preservacionistas. Rev. Geologia..

[25] Alongi D.M. (2012). Carbon sequestration in mangrove forests. Carbon Manag..

[26] Ferreira A.C., Lacerda L.D. (2016). Degradation and conservation of Brazilian mangroves, status and perspectives. Ocean. Coast Manag..

[27] Linares A.P.M., López-Portillo J., Hernández-Santana J.R., Pérez M.A.O., Orozco O.O. (2007). The mangrove communities in the Arroyo Seco deltaic fan, Jalisco, Mexico, and their relation with the geomorphic and physical-geographic zonation. Catena.

[28] Castella R.M.B., Castella P.R., Figueiredo D.C.S., Queiroz S.M.P. (2006). Paraná—Mar e Costa. Subsídios ao Ordenamento das Áreas Estuarina e Costeira do Paraná.

[29] Giri C., Ochieng E., Tieszen L.L., Zhu Z., Singh A., Loveland T., Masek J., Duke N. (2010). Status and distribution of mangrove forests of the world using earth observation satellite data. Glob. Ecol. Biogeogr..

[30] Douhan G.A., Vincenot L., Gryta H., Selosse M.A. (2011). Genética populacional de fungos ectomicorrízicos: Do conhecimento atual às direções emergentes. Biol. Fúngica.

[31] Soares S.C. (1997). Diversity of yeasts in the mangroves of Guanabara Bay and Sepetiba Bay, Rio de Janeiro. J. Appl. Environ. Microbiol..

[32] Oliveira T.B., Lopes V.C.P., Barbosa F.N., Ferro M., Meirelles L.A., Sette L.D., Gomes E., Rodrigues A. (2016). Fungal communities in pressumud compositing harbors beneficial and detrimental fungi for human welfare. Microbiology.

[33] Medrano A. (2021). Taxonomic reclassification of Candida species: A comprehensive review. J. Med. Mycol..

[34] Schlesinger W.H. (1997). Biogeochemistry: An Analysis of Global Change.

[35] Fonseca S.M., Drummond J.A. (2003). Reflorestamento de manguezais e o valor de resgate para o sequestro de carbono atmosférico. Hist. Cienc. Saude Manguinhos.

[36] Grace J. (2001). Carbon cycle. Encyclopedia of Biodiversity.

[37] Niego A.G.T., Rapior S., Thongklang N., Raspé O., Hyde K.D., Mortimer P.D. (2023). Reviewing the contributions of macrofungi to forest ecosystem processes and services. Fungal Biol. Rev..

[38] Read D.J., Perez-Moreno J. (2003). Mycorrhizas and nutrient cycling in ecosystems—A journey towards relevance?. New Phytol..

[39] Bücking H., Mensah J.A., Fellbaum C.R. (2016). Common mycorrhizal networks and their effect on the bargaining power of the fungal partner in the arbuscular mycorrhizal symbiosis. Commun. Integr. Biol..

[40] Chen C., Amirbahman A., Fisher N., Harding G., Lamborg C., Nacci D., Taylor D. (2008). Methylmercury in marine ecosystems: Spatial patterns and processes of production, bioaccumulation, and biomagnification. EcoHealth.

[41] Izzo A., Agbowo J., Bruns T.D. (2005). Detection of plot-level changes in ectomycorrhizal communities across years in an old-growth mixed-conifer forest. New Phytol..

[42] Bellei M.M., Carvalho E.M.S., Cardoso E.J.B.B., Tsai S.M., Neves M.C.P. (1992). Ectomicorrizas. Microbiologia do Solo.

[43] Raven P.H., Evert R.F., Eichhorn S.E. (1996). Biologia Vegetal.

[44] de Souza A.M., de Carvalho D., da Silva S.C., de Lima Pereira N.S. (2001). Caracterização morfológica e isoenzimática de isolados de Pisolithus spp.. Cerne.

[45] Yokomizo N.K.S., Rodrigues E. (1998). Associação ectomicorrízica entre Suillus luteus e Pinus elliottii var. elliottii. Rev. Inst. Florest..

[46] Johnson D., Martin F., Cairney J.W.G., Anderson I.C. (2012). The importance of individuals: Intraspecific diversity of mycorrhizal plants and fungi in ecosystems. New Phytol..

[47] Gusmão L.F.P., Funch L.S., Miranda A.P. (2011). Fungos. Serrano, Parque Municipal da Muritiba.

[48] Kapulnik Y., Waisel Y., Eshel A., Kafkafi U. (1996). Plant growth promotion by rhizosphere bacteria. Plant Roots: The Hidden Half.

[49] Abuzinadah R.A. (1989). Influence of plant growth and nutritional status on ectomycorrhizal fungi. Mycorrhiza.

[50] Siqueira J.O., Franco A.A. (1988). Biotecnologia do Solo: Fundamentos e Perspectivas.

[51] Martin F.M., Öpik M., Dickie I.A. (2024). Mycorrhizal research now: From the micro- to the macro-scale. New Phytol..

[52] Júnior P., Silva R., Oliveira A., Santos M., Almeida T., Costa L., Pereira F., Rodrigues J., Souza D., Lima E. (2021). Micorrizas: Conceitos, Metodologias e Aplicações.

[53] Chancel L. (2022). Desigualdade global de carbono entre 1990 e 2019. Nat. Sustain..

[54] IPCC (2023). Summary for Policymakers. Climate Change 2023: Synthesis Report.

[55] INEP (2014). Adaptation Strategies for Mangrove Ecosystems in the Face of Rising Sea Levels.

[56] Almeida H.S., da Silva R.F., Grolli A.L., Scheid D.L. (2017). Ocorrência e diversidade da fauna edáfica sob diferentes sistemas de uso do solo. Rev. Bras. Tecnol. Agropecu..

[57] Ellison J.C., Stoddart D.R. (1991). Mangrove ecosystem collapse during predicted sea-level rise: Holocene analogues and implications. J. Coast Res..

[58] Wilkin S. (2016). Emerging fungal diseases in mangrove ecosystems. Mar. Ecol. Prog. Ser..

[59] Panackal A. (2011). Global Climate Change and Infectious Diseases: Invasive Mycoses. J. Earth Sci. Clim. Change.

[60] Casadevall A. (2017). Don’t forget the fungi when considering global catastrophic biorisks. Health Secur..

[61] Oliveira A.P. (2007). The presence of mercury in the mangrove ecosystem of Baixada Santista, São Paulo, Brazil. Environ. Pollut. Ecotoxicol. J..

[62] Oliveira M.L.J., Vidal-Torrado P., Otero X.L., Ferreira J.R. (2007). Mercúrio total em solos de manguezais da Baixada Santista e Ilha do Cardoso, Estado de São Paulo. Quím. Nova.

[63] Bayen S., Wurl O., Karuppiah S., Sivasothi N., Lee H.K., Obbard J.P. (2005). Persistent organic pollutants in mangrove food webs in Singapore. Chemosphere.

[64] Guimarães R.R., Silva J.P., Oliveira M.T., Santos A.L., Almeida R.F., Costa L.M., Pereira F.J., Rodrigues D.S., Souza E.M., Lima T.R. (2010). Influence of sewage discharge on pathogen growth and environmental contamination. Water Res..

[65] Sánchez-Quinto A., Costa J.C., Zamboni N.S., Sanches F.H., Principe S.C., Viotto E.V., Casagranda E., da Veiga-Lima F.A., Possamai B., Faroni-Perez L. (2020). Development of a conceptual framework for the management of biodiversity and ecosystem services in the Mexican Caribbean. Biota Neotrop..

[66] Gomes D.N.F., Cavalcanti M.A.Q., Passavante J.Z.O. (2011). Fungos filamentosos isolados de sedimento do manguezal Barra das Jangadas, Jaboatão dos Guararapes, Pernambuco, Brasil. Trop. Oceanogr..

[67] Woodroffe C.D., Robertson A.I., Alongi D.M. (1992). Mangrove sediments and geomorphology. Tropical Mangrove Ecosystems.

[68] Godoy M.D.P., Lacerda L.D. (2014). River-island response to land-use change within the Jaguaribe River, Brazil. J. Coast Res..

[69] Eslami A., Smith J.P., Brown M.T., Davis R.L., Carter G.H., Green P.A., Foster J.R., Hall R.J., Evans K.L., Anderson T.J. (2009). Mangrove migration and climate change: Impacts of sea level rise and sedimentation rates. Glob. Change Biol..

[70] Krauss K.W., Allen J.A., Cahoon D.R., Lynch J.C., Cormier N., Chen R., Twilley R.R., McKee K.L., Lovelock C.E., Saintilan N. (2003). Mangrove forest dynamics in response to environmental change: The role of coastal processes. J. Ecol..

[71] McKee K.L., Cahoon D.R., Feller I.C. (2007). Caribbean mangroves adjust to rising sea level through biotic controls on change in soil elevation. Glob. Ecol. Biogeogr..

[72] Lovelock C.E., Adame M.F., Bennion V., Hayes M., Reef R., Santini M., Cahoon D.R. (2015). Sea level and turbidity controls on mangrove soil surface elevation change. Estuar. Coast. Shelf Sci..

[73] Schleupner C. (2008). Vulnerability of mangrove ecosystems to land-based pollution: A review. Estuar. Coast. Shelf Sci..

[74] Li J., Heath I.B. (1992). The phylogenetic relationships of the anaerobic chytridiomycetous gut fungi (Neocallimasticaceae) and the Chytridiomycota. I. Cladistic analysis of rRNA sequences. Can. J. Bot..

[75] Kulkarni R., Sharma P., Desai A., Patel M., Singh R., Gupta N., Mehta S., Rao K., Joshi V., Nair P. (2018). Pharmaceutical residues and heavy metals in mangrove ecosystems: Impact on biodiversity and ecological functions. Environ. Toxicol. Chem..

[76] Gustin M.S., Amos H.M., Huang J., Miller M.B., Bash J.O., Smith S., Selin N.E., Jaffe D.A., Holmes C.D., Obrist D. (2015). Role of vegetation in the atmospheric deposition of mercury: A review. Environ. Pollut..

[77] Obrist D., Johnson D.W., Lindberg S.E., Luo Y., Hararuk O., Bracho R., Battles J.J., Dail D.B., Edmonds R.L., Monson R.K. (2018). Mercury cycling in the mangrove forests of tropical and subtropical regions. Environ. Toxicol. Chem..

[78] Liu G., Cai Y., Driscoll N.O. (2011). Environmental Chemistry and Toxicology of Mercury.

[79] Driscoll C.T., Mason R.P., Chan H.M., Jacob D.J., Pirrone N. (2013). Mercury as a global pollutant: Sources, pathways, and effects. Sci. Total Environ..

[80] Rice D.C., Schoeny R., Mahaffey K. (2003). Methods and rationale for derivation of a reference dose for methylmercury by the US EPA. Risk Anal. Int. J..

[81] Dorea J., Barbosa A., Ferrari Í., De Souza J. (2003). Mercury in hair and in fish consumed by Riparian women of the Rio Negro, Amazon, Brazil. Int. J. Environ. Health Res..

[82] Patil P.K., Chandra S., Bhat R.A. (2018). Assessment of metal contamination in the Mandovi estuary, Goa. Environ. Pollut..

[83] Lee J.W., Choi H., Hwang U.K., Kang J.C., Kang Y.J., Kim K.I., Kim J.H. (2019). Toxic effects of lead exposure on bioaccumulation, oxidative stress, neurotoxicity, and immune responses in fish: A review. Environ. Toxicol. Pharmacol..

[84] El-Metwally M.A., Abu El-Regal A.I., Abdelkader E.F., Sanad E.F. (2022). Heavy metal accumulation in zooplankton and impact of water quality on its community structure. Arab. J. Geosci..

[85] Dehghani M., Ahmadi S., Hosseini R., Karimi A., Ghasemi N., Mohammadi F., Ebrahimi M., Tavakoli H., Zare M., Shafiei S. (2022). Heavy metal absorption in aquatic organisms: Mechanisms, bioaccumulation, and ecological implications. Environ. Toxicol..

[86] Smith S.E., Read D.J. (2008). Mycorrhizal Symbiosis.

[87] Hagler A.N., Martins L.F., Barros J.P., Santos R.C., Almeida T.M., Costa P.R., Pereira L.A., Rodrigues F.J., Souza D.M., Lima E.R. (2017). The relationship between nutrient pollution and pathogen development in aquatic systems. Sci. Total Environ..

[88] Fortunato J.M., HYPOlITO R., Moura C.L., Nascimento S.C. (2022). Caracterização da contaminação por metais pesados em área de manguezal, município de Santos (SP). Rev. Inst. Geol..

[89] Morales D.V. (2022). Behavioral and Physiological Effects of Heavy Metals on Fish: A Review and Preliminary Results. Master’s Thesis.

[90] Zhang Z.W., Xu X.R., Sun Y.X., Yu S., Chen Y.S., Peng J.X. (2014). Heavy metal and organic contaminants in mangrove ecosystems of China: A review. Environ. Sci. Pollut. Res..

[91] Pinto F.N., Massone C.G., Senez-Mello T., da Silva F.S., Crapez M.A.C. (2022). Interferência da ocupação urbana na distribuição de poluentes orgânicos persistentes em manguezal. Eng. Sanit. Ambient..

[92] Yan Z.Z., Sun X.L., Xu Y., Zhang Q.Q., Li X.Z. (2017). Accumulation and tolerance of mangroves to heavy metals: A review. Environ. Pollut..

[93] da Silva Paes E., Gloaguen T.V., da Conceição Silva H.D.A., Duarte T.S., da Conceição de Almeida M., Costa O.D.V., Bomfim M.R., Santos J.A.G. (2022). Widespread microplastic pollution in mangrove soils of Todos os Santos Bay, northern Brazil. Environ. Res..

[94] Prarat P., Hongsawat P., Chouychai B. (2024). Microplastic occurrence in surface sediments from coastal mangroves in Eastern Thailand: Abundance, characteristics, and ecological risk implications. Reg. Stud. Mar. Sci..

[95] Mohan P., Hamid F.S. (2024). Charting the microplastic menace: A bibliometric analysis of pollution in Malaysian mangroves and polypropylene bioaccumulation assessment in *Anadara granosa*. Mar. Pollut. Bull..

[96] Bayen S., Segovia Estrada E., Juhel G., Lee W.K., Kelly B.C. (2016). Pharmaceutically active compounds and endocrine disrupting chemicals in water, sediments and mollusks in mangrove ecosystems from Singapore. Mar. Pollut. Bull..

[97] Li Y., Li Q., Zhou K., Sun X.L., Zhao L.R., Zhang Y.B. (2016). Occurrence and distribution of the environmental pollutant antibiotics in Gaoqiao mangrove area, China. Chemosphere.

[98] Liu X., Liu Y., Xu J.R., Ren K.J., Meng X.Z. (2016). Tracking aquaculture-derived fluoroquinolones in a mangrove wetland, South China. Environ. Pollut..

[99] Lima S.R., Martins D., Andrade M.C. (2013). Dinâmica hidrológica na Baixada Santista e suas implicações para o planejamento urbano. Rev. Bras. Recur. Hídr..

[100] Noormohamadi S. (2019). Bioremediation of Cd and Ni by *Phanerochaete chrysosporium* in contaminated environments. Environ. Technol..

[101] Munk L., Sitarz A.K., Kalyani D.C., Mikkelsen J.D., Meyer A.S., Jensen P.A., Larsen T., Pedersen S., Hansen K., Nielsen J. (2015). White-rot fungi and their potential for the bioremediation of environmental pollutants. Fungal Biol. Rev..

[102] Jaen-Gil A., Aparicio J., González R., López D., Martínez J., Pérez M., Sánchez A., Torres M., Vidal C., Zamora P. (2019). Fungal bioremediation of pharmaceutical contaminants. Environ. Toxicol. Pharmacol..

[103] Huang Y., Li X., Wang J., Zhang H., Chen L., Liu Y., Zhao W., Zhou Y., Sun Q., Feng J. (2017). Removal of lead (Pb) by *Phanerochaete chrysosporium* and its application in the treatment of wastewater. Chemosphere.

[104] Bhattacharya S., Das A., Ghosh S., Banerjee R., Mukherjee S., Chatterjee T., Roy P., Saha D., Basu S., Dutta P. (2017). Degradation of polycyclic aromatic hydrocarbons by white-rot fungi. Environ. Sci. Pollut. Res..

[105] Wang L., Zhang J., Li H., Chen Y., Liu X., Zhao Q., Sun W., Zhou Z., Yang F., Xu J. (2019). Biodegradation of endocrine-disrupting compounds by white-rot fungi. J. Environ. Sci..

[106] Kaur G., Singh S., Sharma R., Gupta R., Kumar A., Mehta P., Chawla P., Arora S., Dhillon J., Sandhu R. (2016). Fungal degradation of pesticides in contaminated soils. Pestic. Biochem. Physiol..

[107] Hanif M., Bhatti H. (2015). Bioremediation of heavy metals by *Agaricus bitorquis* immobilized in calcium alginate beads. Environ. Sci. Pollut. Res..

[108] Wollenberg M. (2021). Uranium removal by white-rot fungi *Schizophyllum* commune and Pleurotus ostreatus. Environ. Pollut..

[109] Feng M., Yin H., Cao Y., Peng H., Lu G., Liu Z., Dang Z. (2018). Cadmium-induced stress response of *Phanerochaete chrysosporium* during the biodegradation of 2,2’,4,4’-tetrabromodiphenyl ether (BDE-47). Ecotoxicol. Environ. Saf..

